# Diabetic Striatopathy: A Rare Case of Non-ketotic Hyperglycaemia-Induced Hemichorea

**DOI:** 10.7759/cureus.89384

**Published:** 2025-08-04

**Authors:** Shreya Serrao, Duha A Alsheikh, Neelanjana Dutta, Awab Ismail, Mona Mohammednoor

**Affiliations:** 1 Acute and General Medicine, Queen Elizabeth Hospital Birmingham, Birmingham, GBR; 2 Hepatobiliary and Transplant Surgery, Queen Elizabeth Hospital Birmingham, Birmingham, GBR; 3 Internal Medicine, Queen Elizabeth Hospital Birmingham, Birmingham, GBR; 4 General Medicine, Queen Elizabeth Hospital Birmingham, Birmingham, GBR

**Keywords:** chorea hyperglycaemia basal ganglia syndrome, diabetes mellitus, diabetic chorea, diabetic hemichorea, non-ketotic hyperglycaemia

## Abstract

Diabetes mellitus is a metabolic condition leading to elevated blood glucose levels due to insulin deficiency, insulin resistance, or a combination of both. Chronically raised blood glucose levels can lead to a broad variety of microvascular and macrovascular complications. Neurological disorders are a common manifestation of diabetes mellitus, and poorly controlled diabetes mellitus frequently causes peripheral sensorimotor polyneuropathy and autonomic neuropathy. More rare manifestations include diabetic amyotrophy and mononeuritis multiplex. Hemichorea-hemiballismus (HCHB) syndrome is a movement disorder in which there is involuntary, high amplitude, low frequency, and irregular movements involving one side of the body and sparing the other. It is a rare condition, and there are many causes such as metabolic, vascular, neoplastic, infective, demyelinating, and traumatic precipitating causes. This report discusses a 63-year-old female who presented with a one-day history of involuntary movements of her left upper and lower limb. On presentation, she was noted to have high-amplitude, low-frequency movements of her left arm and, to a lesser extent, her leg and was significantly hyperglycaemic on presentation. She had a glycated haemoglobin (HbA1c) level of 144 millimole per mol (mmol/mol) and computerised tomography, and magnetic resonance imaging scans of her brain did not identify any abnormalities in the basal ganglia. On control of her blood glucose levels to lower ranges, her involuntary movements resolved, demonstrating a diagnosis of non-ketotic hyperglycaemic hemichorea.

## Introduction

Non-ketotic hyperglycaemic hemichorea-hemiballismus (HCHB) is a rare movement disorder that occurs in older patients with poorly controlled diabetes. HCHB is characterized by involuntary, irregular, and often violent movements on one side of the body [1,2]. While HCHB is commonly associated with structural lesions in the contralateral basal ganglia, such as those caused by strokes or tumours, recent findings suggest that non-ketotic hyperglycaemia can also trigger HCHB, particularly in older adults with poorly managed diabetes [2].

Recognizing hyperglycaemia-induced HCHB is crucial, as it presents an important opportunity for intervention. Although this condition is not frequently observed in clinical practice, it is a significant neurological complication of uncontrolled diabetes [2].

The exact pathophysiological mechanism remains under investigation, but several theories propose that factors such as hyperviscosity-induced ischemic damage, dysfunction of the blood-brain barrier, and reduced availability of inhibitory neurotransmitters like gamma-aminobutyric acid (GABA) in the basal ganglia may contribute to its development. Neuroimaging studies typically reveal characteristic hyperdensities in the striatum on computed tomography (CT) or T1-weighted hyperintensities on magnetic resonance imaging (MRI), which can aid in diagnosis [3].

Timely recognition of HCHB is essential, as early correction of hyperglycaemia has been shown to lead to significant improvement in symptoms, enabling a more focused and effective treatment approach, as demonstrated in previous case reports [4].

In this report, we present a case of acute-onset HCHB in a patient with uncontrolled type 2 diabetes mellitus, highlighting the clinical presentation, neuroimaging features, and favourable response to glycaemic control.

## Case presentation

This case reports the presentation of a 63-year-old female patient with a background of type 2 diabetes mellitus diagnosed in 2008 when she required inpatient admission and treatment for the hyperglycaemic hyperosmolar state, who presented with a one-day history of involuntary sudden movements affecting her left arm. She had previously been diagnosed with temporal lobe epilepsy 18 years prior to presentation and was managed with carbamazepine. The semiology of her previous seizures were vacant episodes where she stared blankly and were preceded by a visual aura (seeing strange colours) and she had been seizure-free for 12 years. She had not been under Neurology follow-up. For her diabetes mellitus, she was taking metformin, empagliflozin, and gliclazide. Her prior glycated haemoglobin level five months prior to presentation was 66 millimole per mole (mmol/mol), and her body mass index (BMI) was 44.92 kilograms per square meter on presentation.

Prior to the involuntary movements, she had noted herself to have developed a sore throat and dry cough. She had fatigue and increased frequency of micturition. She had therefore stayed at home for a prolonged period. One day prior to presentation whilst sat at home in her living room, she noticed sudden-onset involuntary movements of her left arm. There was no preceding aura and the movements were irregular, low frequency, and large amplitude and caused her to be unable to hold anything due to the hyperkinetic movements, although her strength was preserved. She also noticed irregular involuntary movements of her left leg. These movements continued until her presentation to hospital the following day.

**Figure 1:**
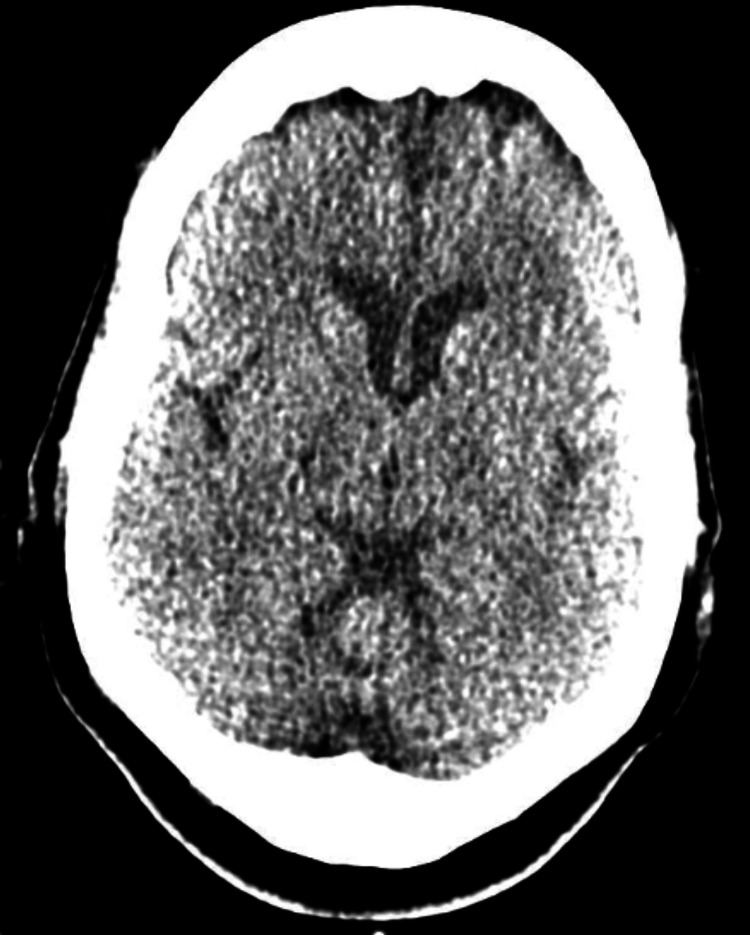
Non-contrast CT brain with no remarkable identified pathology.

On initial assessment, she was alert and oriented to time, place, and person. She had a Glasgow Coma Score (GCS) of 15/15. She had witnessed high-amplitude, low-frequency, irregular, and involuntary movements of her left upper limb. Her capillary blood sugar level was above 33 millimoles per litre (mmol/L), and her serum ketone levels were 0.3 mmol/L. She subsequently had a venous blood gas sample taken, and the results showed a pH of 7.349 and a bicarbonate level of 23.1 mmol/L. She had a serum sodium concentration of 120 mmol/L (Tables 1, 2). Unfortunately, no serum osmolality was sent at that time prior to correction of the hyponatraemia. She subsequently had a plain CT scan of her brain (Figure 1), which was reported as showing increased attenuation of the right basal ganglia (although these findings could not be clearly identified on the imaging as shown). A CT angiography of her intracranial and extracranial vessels was also performed, which did not show any vessel occlusion or other remarkable finding.

**Table 1:** Patient laboratory results compared with normal expected values

Laboratory test	Patient value	Normal range	Units
Full blood count			
Haemoglobin (Hgb)	129	115-154	g/L
White blood cell (WBC) count	7.19	3.00-10.90	x109 cells/µL
Platelet count	195	150-400	x109 /L
Neutrophils	4.6	1.50-7.10	x109 /L
Lymphocytes	1.9	0.60-4.00	x109 /L
Monocytes	0.51	0.24-0.90	x109 /L
Eosinophils	0.08	0.03-0.51	x109 /L
Mean corpuscular volume (MCV)	86.2	81.0-102.0	fL
Mean corpuscular haemoglobin (MCH)	29.3	27-34	PG
Mean corpuscular haemoglobin concentration (MCHC)	339	298-340	g/L
Nucleated red blood cell (NRBC)	0.00	0-0.01	x109 /L
Haematocrit (HCT)	0.380	0.350-0.480	L/L
International normalised ratio (INR)	1.0	0.8-1.2	INR
C-reactive protein (CRP)	15	<0.5	mg/L
Glycated haemoglobin (HbA1c)	148	20-42	mmol/mol
Urea and electrolytes			
Sodium (Na)	119	133-146	mmol/L
Potassium (K)	5.5	3.5-5.3	mmol/L
Urea	13.8	2.5-7.8	mmol/L
Creatinine	106	49-90	umol/L
eGFR (estimated glomerular filtration rate)	48	>90	mL/min
Magnesium (Mg)	1.17	0.70-1.00	mmol/L
Phosphate (PO4)	1.13	0.80-1.50	mmol/L
Corrected calcium	2.29	2.20-2.60	mmol/L
Liver function test			
Albumin	39	35-50	g/L
Total protein	74	60-80	g/L
Bilirubin	5	<21	umol/L
Alanine transaminase (ALT)	29	0-55	U/L
Alkaline phosphatase (ALP)	190	30-130	U/L
Thyroid function test (TFT)			
Free T4	11.5	9.0-19.0	pmol/L
TSH	1.60	0.40-4.90	mIU/L
Lipid profile			
Cholesterol	6.7	<5.0	mmol/L
Triglyceride	11.60	0.0-1.7	mmol/L
HDL	0.92	>1.55	mmol/L
Haematinics			
B12	615	187-883	ng/L
Folate	6.80	3.10-20.50	ug/L
Iron studies			
Iron	10.7	9.0-30.4	umol/L
Transferrin	2.15	1.73-3.60	g/L
Ferritin	162	20-235	ug/L

**Table 2:** Venous blood gas findings on presentation compared with normal reference ranges

Laboratory test	Patient value	Normal range	Units
pH	7.349	7.350-7.450	
pO2 (partial pressure of oxygen)	6.2	4.27-6.41	kPa
pCO2 (partial pressure of carbon dioxide)	6.1	11.07- 14.40	kPa
Standard bicarbonate (HCO3)	23.1	22-29	mmol/L
Base excess	-1.3	-2 to +2	mmol/L
Anion gap	14.5	8 to 16	mmol/L
Sodium (Na)	120.4	136-145	mmol/L
Potassium (K)	5.31	3.50-5.10	mmol/L
Ca ion	1.04	1.150-1.330	mmol/L
Chloride	86.6	98-107	mmol/L
Total haemoglobin	130.50	115-178	g/L
SO2	84.2	91-98	%
HCT	51.7	36-53	%
COHb (carboxy-haemoglobin)	2.3	0.0- 3.0	%
MetHb (methaemoglobin)	0.6	0.0-1.5	%
Glucose	NA (Above the upper detection range)	4.10-5.60	mmol/L
Lactate	2.68	0.20-1.80	mmol/L

She was subsequently commenced on a variable rate insulin infusion (VRII). Her empagliflozin was paused due to the risk of ketosis, and a repeat HbA1c blood test was obtained. An inpatient magnetic resonance imaging (MRI) scan of her brain was requested, and she was commenced on insulin glargine 22 units once daily. Her HbA1c result was 144 mmol/mol, and the subsequent management plan was to establish insulin therapy and for inpatient insulin education prior to discharge.

**Figure 2:**
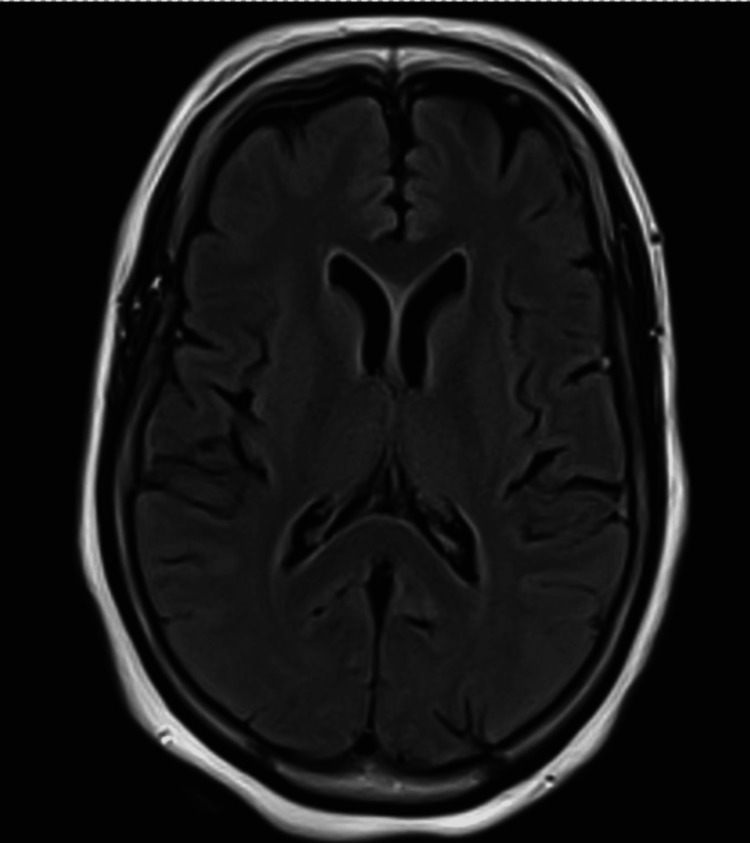
FLAIR image MRI brain, axial view, with no demonstrable significant findings. FLAIR: fluid-attenuated inversion recovery, MRI: magnetic resonance imaging

**Figure 3:**
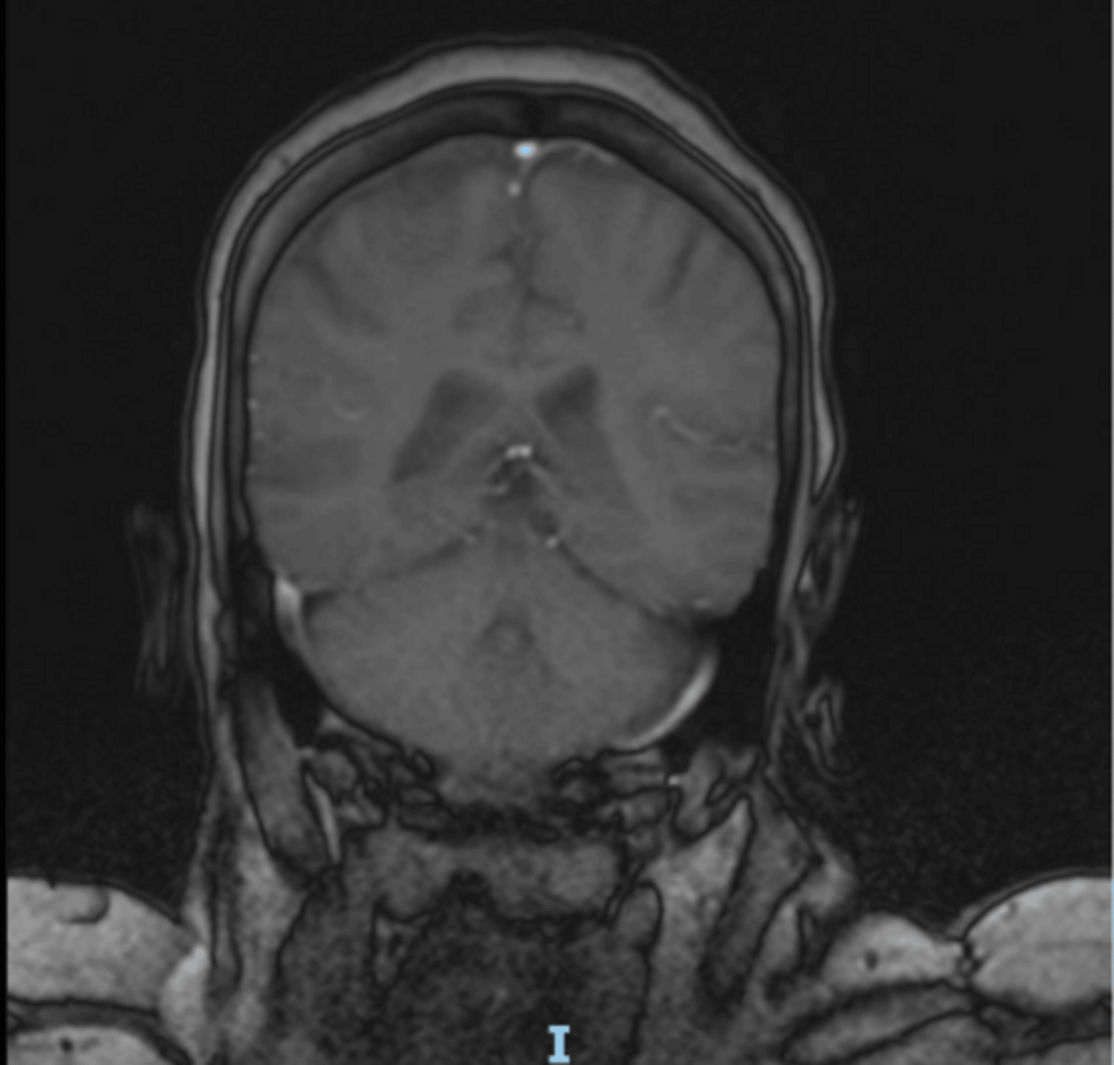
T1-weighted MRI brain with contrast in the coronal view with the basal ganglia in view

Her subsequent MRI brain scan (Figures 2, 3) report showed normal appearance and signal return from the basal ganglia with only a single non-specific hyperintense focus on the right frontal lesion of the subcortical white matter.

Following nine hours of treatment with variable rate insulin, the patient’s involuntary movements had stopped. At this time, her blood glucose levels had reduced from undetectably high above 33 mmol/L to 28.4mmol/L. Her sodium levels improved and were thought to be pseudo-hyponatraemia in the context of raised plasma glucose levels. 

Following on from this, she was switched onto Humalog 50 at 40 units three times daily due to patient preference of reducing daily injections. Her blood glucose levels were stable once on an established dose, and she received insulin education and was discharged, with Diabetes Clinic follow-up planned. 

## Discussion

A wide variety of neurological manifestations can be expected in diabetes mellitus, the pathophysiology of which has been linked to micro-angiopathy and macro-angiopathy related to poor glycaemic control, as outlined in the Oxford Handbook of Endocrinology and Diabetes [5]. Diabetic striatopathy (DS), also known as non-ketotic hyperglycaemic hemichorea is well-recognised in previous literature, including a large retrospective study performed in Italy by Ottaviani et al. [6]. In that study, it was defined as a state of hyperglycaemia associated with chorea/ballism, striatal hyperdensity at CT, or hyperintensity at T1-weighted MRI [6]. Furthermore, Ottaviani et al. demonstrated in that retrospective study the extremely rare prevalence of the condition, with only three patients in the study diagnosed with it out of a population of 1,806 neuro-imaged patients with poorly controlled diabetes mellitus (HbA1c more than 64 mmol/mol). 

The condition has been reported in younger age groups in type 1 diabetes mellitus, such as in a case report by Kartik V et al. in which a 20-year-old male patient presented with hemichorea linked to significant hyperglycaemia [7]. In that particular case, the neuroimaging showed the classical hyperattenuation in the contralateral basal ganglia. In comparison, the case highlighted in this report had more subtle findings on MRI and non-contrast CT (Figures 1, 2, 3), but in both cases, symptoms improved rapidly with improvement in the blood glucose levels. The more subtle neuroimaging findings in the case outlined in this report highlights that clinical diagnosis remains paramount, particularly in cases of normal radiological findings. 

In terms of other metabolic causes of chorea, StatPearl provides a comprehensive review of these and their prevalence [8]. Commonly implicated metabolic causes outlined in that tertiary-source online reference include hepatic failure, renal failure, electrolyte abnormalities, and thyroid disorders. The absence of ketones and a normal pH (7.349) confirmed a non-ketotic state. The absence of electrolyte abnormalities, deranged liver enzymes, and/or renal function on laboratory investigations, distinguishing it from other metabolic encephalopathies [8]. 

Moreover, this patient's significantly elevated HbA1c (144 mmol/mol) pointed toward chronic poor glycaemic control, correlating with the severity of her presentation. 

The history of epilepsy in this patient added a layer of complexity. While she had not had any seizures in over a decade, it was crucial to differentiate these new symptoms from a possible relapse. The lack of aura, the specific type of movements, and the absence of post-ictal confusion helped to rule out a seizure, as these are all usual manifestations of an epileptic seizure [1]. 

Clinically, her involuntary movements and high-amplitude, low-frequency, irregular movements were consistent with HCHB as portrayed in numerous similar cases highlighted in the literature [2,3,4,7]. The rapid resolution of involuntary movements within nine hours supports the diagnosis of DS. This also reinforces the reversibility of symptoms with timely metabolic correction as demonstrated in other similar case reports such as the one by Padmanabhan et al. [2]. 

In our outlined case, a variable-rate insulin infusion led to complete resolution of symptoms, which was sustained upon transition to subcutaneous insulin. The sodium levels in this patient were initially low, which was corrected without specific intervention, reflecting pseudo-hyponatraemia due to hyperglycaemia, a common artefact in such metabolic disturbances [5]. 

The patient’s preference for premixed insulin (Humalog 50) with fewer daily injections underscores the importance of individualised diabetes management and patient-centred care [5]. Furthermore, comprehensive diabetes education and ongoing outpatient care are vital for preventing recurrence of hyperglycaemia. 

In the retrospective study by Ottaviani et al. into the prevalence of this rare condition, it was shown that it is relatively more common among the Asian population [6]. However, in that study, there were only three patients confirmed to have the condition, so these data cannot be relatively relied upon in terms of demographic distribution. Interestingly, the patient in this case report was Afro-Caribbean, which suggests that DS is not exclusive to any one ethnicity. This suggests that future research is warranted to better understand the pathophysiological mechanisms of the syndrome, particularly in relation to ethnicity. 

This case adds to the growing literature of DS, highlighting the fact that glycaemic control is not only therapeutic but also diagnostic. For clinicians, especially those in emergency and acute medical settings, recognising hemichorea as a possible complication of hyperglycaemia can prevent unnecessary tests and lead to prompt, effective treatment. Although rare, DS is highly treatable and often reversible if caught early and managed appropriately. 

## Conclusions

This case presentation highlighted a rare manifestation of poor glycaemic control and non-ketotic hyperglycaemia. It demonstrated the importance of looking into metabolic causes for hemichorea and management of these reversible causes. The confounding complicating factors in this case, such as the formal previous diagnosis of epilepsy and the significantly elevated HbA1c on presentation, indicating that she had poor long-standing glycaemic control, both made this an atypical presentation. However, the rapid response to tight glycaemic control aligned with the literature and solidified the diagnosis, particularly in the presence of subtle characteristic basal ganglia changes on neuroimaging. Future research into the demographics, co-existing epilepsy, and HbA1c at presentation of similar cases would improve our understanding and recognition of diabetes-associated striatopathy.
